# Correlation of Clinical and Imaging Findings in Early‐Onset Alzheimer's Patients with Distinct Initial Symptoms

**DOI:** 10.1002/alz.094057

**Published:** 2025-01-09

**Authors:** Sinem Özçelik, Pınar Yiğit, Tolga Can Bozdemir, Gamze Çapa Kaya, Zerrin Yildirim, Boon Lead Tee, Hakan Gurvit, Görsev Yener, Maria Luisa Gorno Tempini, Didem Öz

**Affiliations:** ^1^ Dokuz Eylul University Hospital Neurology Department, Izmir Turkey; ^2^ Dokuz Eylul University Graduate School of Health Sciences, Izmir Turkey; ^3^ Dokuz Eylul University Hospital Department of Nuclear Medicine, Izmir Turkey; ^4^ Neuroimaging Laboratory, Hulusi Behcet Life Sciences Research Center, Istanbul University, Istanbul Turkey; ^5^ Memory and Aging Center, UCSF Weill Institute for Neurosciences, University of California, San Francisco, San Francisco, CA USA; ^6^ Behavioural Neurology and Movement Disorders Unit, Department of Neurology, Istanbul Faculty of Medicine, Istanbul University, Istanbul, Turkey, Istanbul Turkey; ^7^ Izmir Biomedicine and Genome Center, Izmir Turkey; ^8^ Memory & Aging Center, Department of Neurology, University of California in San Francisco, San Francisco, CA USA; ^9^ GBHI ‐ UCSF, San Francisco, CA USA

## Abstract

**Background:**

Early‐onset Alzheimer’s disease (EOAD), distinct from late‐onset cases, tends to present with atypical cognitive decline, such as, progressive aphasia, apraxia, visuospatial and comportmental impairments,. This study aims to investigate correlations between clinical presentation and PET imaging findings in EOAD patients.

**Method:**

A retrospective analysis was conducted on CSF confirmed EOAD patients. Clinical assessments incorporated ACE‐R cognitive tests, MRI and FDG‐18 PET CT. MRIs were rated by an experienced neurologist using the Visual Atrophy Scale (VAS). Patients were stratified based on clinical presentation, and correlations between ACE‐R domains and PET indices were examined. FDG‐18 PET index was calculated as the count number (CN) of the same regions evaluated in VAS divided by CN of their cerebellum.

**Result:**

Nineteen patients (57.9% women) with a mean age at disease onset 55.7 (±6.57 StD) years participated. The mean disease duration was 5.1 (±3.14 StD) years. In the typical amnestic group (n=8); ACE‐R total scores (52.63±15.3 StD) exhibited correlations with left parietal (r: 0.775, p: 0.025) and left occipital PET indices (r: 0.825, p: 0.012). Moreover, total memory scores (9.13±7.4 StD) correlated with left anterior temporal (r: 0.848, p: 0.008), and repetition scores correlated with left parietal PET indices (r: 0.907, p: 0.005). In the amnestic subgroup (n=5), a notable correlation was found between retrograde memory scores and left anterior temporal PET index (r: 0.905, p: 0.035). In the visuospatial group (n=8) orientation scores with left anterior temporal (r: 0.092, p: 0.02), and writing scores with right occipital (r: 0.968, p: 0.07) and left parietal (r: 0.941, p: 0.017) was found correlated. The behavioral variant group (n=3) showed a correlation with right orbitofrontal PET activity (r: 0.99, p: 0.02). A significant difference in attention scores was noted between aphasia and amnestic groups (p = 0.032), with the amnestic group scoring higher (6.20 (4.50‐10.50); 16.44 (11.50‐17.75)).

**Conclusion:**

Distinct correlations emerged between cognitive domains and PET activity based on early symptoms in EOAD patients, emphasizing the intricate heterogeneity within EOAD. The findings offer valuable insights for personalized interventions and advance our understanding of the multifaceted manifestations in the EOAD spectrum.

